# Correlates of Parental Consent to Human Papillomavirus Vaccine Uptake by Their Adolescent Daughters in ZAMBIA: Application of the Health Belief Model

**DOI:** 10.3390/vaccines11050912

**Published:** 2023-04-28

**Authors:** Mwansa Ketty Lubeya, Carla J. Chibwesha, Mulindi Mwanahamuntu, Moses Mukosha, Innocent Maposa, Mary Kawonga

**Affiliations:** 1 Department of Obstetrics and Gynaecology, School of Medicine, The University of Zambia, Lusaka 10101, Zambia; mulindim@gmail.com; 2 Women and Newborn Hospital, University Teaching Hospitals, Lusaka 10101, Zambia; 3 School of Public Health, Faculty of Health Sciences, University of the Witwatersrand, Johannesburg 2193, South Africa; mukoshamoses@yahoo.com (M.M.); mary.kawonga@wits.ac.za (M.K.); 4 Clinical HIV Research Unit, Helen Joseph Hospital, Johannesburg 2193, South Africa; carla_chibwesha@med.unc.edu; 5 Department of Pharmacy, School of Health Sciences, University of Zambia, Lusaka 10101, Zambia; 6 Department of Epidemiology and Biostatistics, School of Public Health, Faculty of Health Sciences, Witwatersrand University, Johannesburg 2193, South Africa; innocent.maposa@wits.ac.za; 7 Department of Community Health, Charlotte Maxeke Johannesburg Academic Hospital, Johannesburg 2193, South Africa

**Keywords:** human papillomavirus vaccination, HPV vaccine, human papillomavirus, genital warts, knowledge, mediation analysis, socio-economic status, cervical cancer screening, generalised structural equation modelling, sub-Saharan Africa

## Abstract

Parental consent for adolescent human papillomavirus (HPV) vaccine uptake is important; however, refusal is prevalent. Therefore, this study aimed to understand factors associated with parental consent for their adolescent daughter’s HPV vaccination. A cross-sectional study was conducted in Lusaka, Zambia, between September and October 2021. We recruited parents from different social settings. The means and standard deviations or median and interquartile ranges were used as appropriate to summarise continuous variables. Simple and multiple logistic regression models were fitted with robust estimation of standard errors. The odds ratios are presented with 95% CI. Mediation analysis was conducted using a generalised structural equation model. The study enrolled 400 parents, mean age 45.7 years [95% CI, 44.3–47.1]. Two hundred and fifteen (53.8%) parents reported consenting to their daughters’ HPV vaccination, and their daughters received it. None of the health belief model (HBM) construct scores showed an independent association with parental consent. Higher, compared to lower wealth index (AOR; 2.32, 95% CI: 1.29–4.16), knowing someone with genital warts (AOR = 2.23, 95 CI: 1.04–4.76), cervical cancer screening uptake (AOR = 1.93, 95% CI: 1.03–3.62) were associated with increased odds of parental consent. This study highlights factors influencing parental consent for their daughters’ HPV vaccination. Ongoing sensitisation programs are important to improve their decision-making.

## 1. Introduction

The human papillomavirus (HPV) vaccine is one of the greatest inventions in preventing HPV-related conditions. HPV is sexually transmitted, with a significant proportion of sexually active people getting infected in their lifetime [1]. Infection transmission is highest during sexual debut [1]. The point prevalence of HPV infection is estimated at 11–12% globally and 22–24% for sub-Sahara Africa (SSA) [2]. The World Health Organisation (WHO) prequalified the use of the HPV vaccine among adolescent girls in 2006 since it is more efficacious if administered before sexual debut [3]. From inception, the number of vaccine manufacturers was limited; however, manufacturers from China (bivalent Cecolin^®^, Xiamen Innovax Biotech Co., Ltd., Xiamen, China) and India [a quadrivalent vaccine from Serum Institute of India-pending prequalification] have come on board in a bid to improve the supply [4,5]. The available HPV vaccines offer protection against prespecified strains; however, cross-protection does occur [6]. HPV vaccines currently on the market include the bivalent (16, 18), quadrivalent and (6, 11, 16, 18) nine-valent (6, 11, 16, 18, 31, 33, 45, 52, 58) [7].

The HPV vaccine is safe and effective for the primary prevention of cervical cancer, which has high morbidity and mortality in sub-Saharan Africa (SSA) [8]. Studies have also shown that the HPV vaccine could be therapeutic in women already infected with high-risk types of HPV 16, 18 and 31 [9]. The World Health Organisations’ global strategy to eliminate cervical cancer is set to be operationalised by achieving three targets by 2030 which are; (i) 90% of girls aged 15 years are fully vaccinated against HPV, (ii) 70% of women of reproductive age screened for cervical cancer at least twice at 35 and 45 years with a high-performance test (iii) 90% of women with pre/cancers receive appropriate treatment [10]. Therefore, it is prudent to deliberate nationally to work towards these targets. The primary focus of this paper is on the first target of the global strategy on the use of HPV vaccines among adolescent girls for cervical cancer prevention in Zambia.

Zambia has one of the world’s highest cervical cancer incidence rates of 65.5 per 100,000 women and a mortality of 43.3 per 10,000 women, accounting for 23% of all new cancers in the country in 2020 [11,12]. With this high incidence, Zambia is among the first countries in SSA to have introduced a free national cervical cancer screening program, initially targeting women living with human immunodeficiency (WLHIV), later including HIV-negative counterparts [13,14], however screening uptake remains low [15]. Co-infection with HIV and HPV increases the persistence of high-risk HPV infections, rendering WLHIV at an increased risk of developing cervical cancer [14,16]. Furthermore, Zambian women of reproductive age suffer a disproportionately higher incidence of HIV than their male counterparts, increasing the risk of contracting cervical cancer [17]. Literature has shown that WLHIV are up to six times more likely to have cervical cancer than women without HIV [18].

To complement the efforts made through cervical cancer screening, Zambia introduced a campaign-based free national HPV vaccination program in 2019 restricted to a single age cohort of 14-year-old girls with a two-dose regimen, 12 months apart [17]. However, there are challenges of low HPV vaccine uptake; for example, dose one coverage was 78% in 2019, reducing drastically to 39% in 2021 [19]. Some of the reasons implicated in this low uptake include beliefs in myths and misinformation about the HPV vaccine, such as; it can cause infertility, HPV vaccination is viewed as a process of initiation into satanism, school closures due to the COVID-19 pandemic hence schools not accessible as a delivery platform, belief in myths around the COVID-19 vaccine, poor social mobilisation, parents’ lack of awareness and non-willingness to consent for daughters’ vaccination [19,20].

HPV vaccination in Zambia is non-mandatory; parental consent is required with or without the parent’s presence at the point of vaccination. WHO has guided the consent process for eligible adolescents to be reviewed according to country guidelines; hence informed parental consent may be written, verbal or implied [21]. Parental consent plays a critical role in the HPV vaccination process for adolescent girls deemed too young to consent for themselves. Although the HPV vaccine is highly efficacious and safe, vaccine refusal among parents of adolescents is prevalent [22].

Since its national rollout, there has been a paucity of data on factors influencing parental HPV vaccination consent in the Zambian context. In addition, mechanisms that potentially drive parental HPV vaccination consent have not been explored. Previously, Lui et al. [23] reported high HPV vaccine acceptance among female parents in a hypothetical study done in Lusaka, Zambia, before the national rollout. However, this did not match the current low uptake of 39% in 2021, for example [19]. Other regional studies have shown that even when girls are eligible, they do not receive the HPV vaccine due to non-consent by their parents, posing a considerable barrier to vaccination [24].

Understanding factors influencing parental consent to HPV vaccination, such as beliefs, knowledge, and attitudes, is critical in developing targeted strategies to increase HPV vaccine uptake among adolescent girls [25,26]. Therefore, to understand parents’ beliefs and attitudes that influence consenting to HPV vaccine uptake for daughters, we used the health belief model (HBM) as a guiding framework. The HBM is a cognitive model with six constructs: perceived susceptibility, perceived severity, perceived benefits, perceived barriers, self-efficacy, and cues to action [27]. The HBM has been used extensively in health behaviour research to explain the uptake of health interventions such as HPV vaccination uptake [28,29,30], COVID-19 vaccination hesitancy [31] and cervical screening uptake [32]. However, there are conflicting results in the literature, possibly due to contextual variations such as geographical location, cultural aspects, sample size and study design [33]. We, therefore, included in our analysis other factors such as socio-demographic characteristics, HPV & HPV vaccine knowledge, cervical cancer screening and HIV status, which have been found to predict HPV vaccine consent behaviours [28].

**Figure 1:**
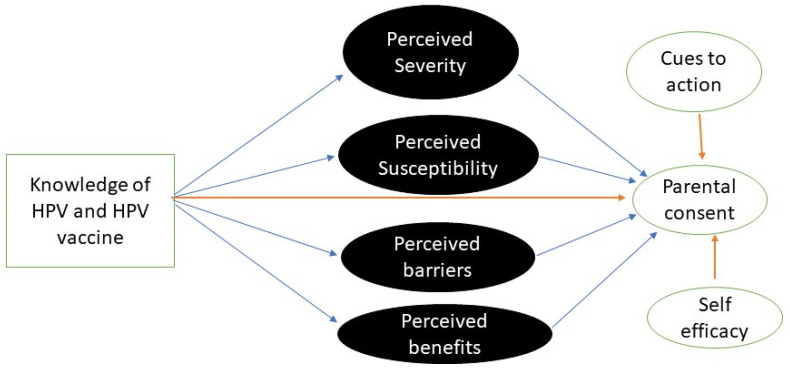
Knowledge directly (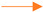) affecting parental consent to vaccinate their daughter or mediated indirectly (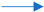) by 4 HBM constructs. At the same time, the other 2 HBM constructs directly affecting (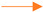) the outcome.

To our knowledge, HBM has not been used previously in Zambia in the context of parental consent for HPV vaccination. Therefore, we hypothesised that parents with higher perceived severity, perceived benefits, perceived susceptibility, higher self-efficacy, more cues to action and lower perceived barriers would be more likely to consent to their daughters’ HPV vaccination [29]. Further, based on the findings from earlier studies in Zambia [34,35], we hypothesised that parents with higher knowledge of HPV and HPV vaccine would be more likely to consent to their daughter’s vaccination. Lastly, we hypothesized that the effect of HPV and HPV vaccine knowledge levels on parental consent might be mediated by different HBM constructs, as shown in the conceptual diagram in Figure 1.

## 2. Materials and Methods

### 2.1. Study Design and Setting

We conducted a cross-sectional study between September and October 2021, in Lusaka district, the capital city of Zambia, with a population of 1,747,152 million people, 417.9 km^2^ area and a population density of 4181/km^2^ [36]. The district is divided into six subdistricts: Chilenje, Chawama, Chelston, Chipata, Matero and Kanyama. Primary healthcare facilities within the subdistricts have the task of offering the HPV vaccine to adolescent girls based on the catchment population. During the 2019 vaccine rollout program, 331,154 teenage girls (14 years old) were eligible for dose one and 212,509 in 2020 and 420,704 in 2021 [19].

The target number of adolescent girls for HPV vaccination is determined using school registers from the Ministry of General Education, headcount, and Central Statistical Office figures. Community health workers and civil societies additionally identify out-of-school girls. The HPV vaccination program is campaign based, conducted during the first round of child health week (CHWk1), lasting about six days from Monday to Saturday. The CHWk1 is a biannual event that aims to catch up on vaccinations for children under five years from health facilities and outreach/mobile sites. Additionally, for HPV vaccination, schools are used, making it a mixed approach to capture both in-school and out-of-school girls.

The district health director and health facilities work closely with the Ministry of General Education and schools in planning. Vaccination dates are shared with the school by the district health team so that teachers can prepare for the activity and girls can inform their parents in addition to community and mass media messages. Information sharing with parents varies, ranging from flyers, educational materials, television adverts, radio programs and letters.

The parental consent approach is opt-out, meaning consent is implied unless a girl categorically mentions that her guardians refused; most private schools, however, use the opt-in approach, which requires written parental consent before vaccination [37]. However, if parents decline and the girl is willing to be vaccinated, her wish supersedes parental consent, and the vaccine is given. Healthcare workers play the role of administering the vaccine and educating different stakeholders, whereas teachers play the role of registering and organising the eligible girls, including informing parents. Girls living with HIV take the first HPV vaccine dose with their peers, the intermediate dose from the health facility during the routine anti-retroviral therapy clinics, and the third dose at 12 months when their peers receive the second dose.

### 2.2. Sampling and Sample Size Considerations

Based on similar studies’ estimates [28], we assumed that 267 participants would be sufficient to estimate the proportion of parents who would consent to vaccinate their daughters with precision ±5%. In addition, the sample gave us over 80% power to detect an absolute difference of 20% between consenting and non-consenting parents to vaccinate their daughters as statistically significant at the 5% level between these two equally sized groups.

A two-stage sampling technique was used to enroll respondents. In the first stage, we stratified Lusaka into six sub-districts. Then we randomly selected two zones in each sub-district using probability proportion to zone size. In the second stage, a random sample of 30 respondents per zone was drawn from various social settings, such as markets, saloons, and barber shops, until we reached 400 participants clustered within 12 zones. We inflated the final sample size to 400 participants after accounting for the assumed moderate design effect of 1.5 to counter the loss in precision caused by the clustering of respondents in the 12 zones.

### 2.3. Data Collection and Variables

Face and content validity of the questionnaire was ascertained by Obstetrician/Gynaecology consultants through a consultative process. We developed an interviewer-administered questionnaire based on the literature [29,38]. The questionnaire was developed in English and translated into one local language (Chinyanja) by a qualified local translator. We did not back-translate the questionnaire at analysis since all the questions were quantitative. We piloted the questionnaire among 33 participants in three sub-districts of Lusaka who were later excluded from the final analysis.

A screening question was asked if the potential participant had a daughter aged 15–18 years. If not, the interview was terminated. This age group was selected because the HPV vaccination program in Zambia targets 14-year-old adolescent girls for dose 1. Therefore, we anticipated that an age range of 15–18 years would give a sample of parents with daughters who would have been eligible to receive the first dose or completed the vaccination schedule during the HPV vaccination demonstration (2013–2017) [20,39] or national roll-out (2019) [40] HPV vaccination programs.

The outcome variable was parental consent for the HPV vaccine for daughters measured on a binary scale (yes = 1, no = 0). The primary exposure variable was the HBM constructs scores. The total scores for the HBM constructs were calculated using the following steps: For each item within the HBM constructs, the numeric values chosen by each respondent, with a five-point Likert scale, were added. Responses were coded such that higher scores indicate a greater level of the relevant construct (strongly disagree = 1, disagree = 2, neutral = 3, agree = 4, strongly agree = 5) and the reverse for perceived barriers. The total possible scores per construct were as follows; (a) perceived susceptibility (3 items = 15), (b) perceived severity (4 items = 20), (c) perceived benefits (4 items = 20), (d) perceived barriers (8 items = 40), (e) self-efficacy (4 items = 20), and (f) cues to action (2 items = 10). Knowledge of HPV (18 items) and HPV vaccine (24 items) scale had three options (true/false/don’t know). We assigned a zero for “false/don’t know” and a one for “true” options.

In addition, the study measured HPV and HIV-related parental characteristics, including ever screening for cervical cancer (females) (yes = 1, no = 0), parental HIV status (negative = 0, positive = 1, prefer not to say/don’t know = 3), the daughter’s HIV status (negative = 0, positive = 1, prefer not to say/don’t know = 3), and knowing someone with cervical cancer or other HPV-related cancers (yes = 1, no = 0), Socio-demographics included age (continuous), sex (male = 0, female = 1), marital status (single = 0, married = 1) employment(yes = 1, no = 0) and socio-economic status (wealth index as proxy).

The socio-economic status variables used for calculating the wealth index included; source of drinking water, type of toilet, house ownership, employing anyone, use of electricity, type of cooking fuel used and ownership of any of the following; fridge, laptop, television, motorcycle, scooter, mobile phone, watch, livestock, land phone, car, agricultural land, washing machine [41].

Data collectors were trained over five days to understand the questionnaire, the study protocol and the use and configuration of the Open Data Kit (ODK) for data collection (https://getodk.org/). ODK is a free mobile-based platform with four tools serving different functions, namely, Aggregate, Build, Collect and Voice [42]. We used ODK Collect, which can run on Android-based devices and supports multiple methods of transforming data and other services for online and offline use. Further, ODK collect allows building forms in multiple languages, which can be used interchangeably within and between participants with a button click [42]. Due to its multiple advantages, ODK has been widely used in HPV vaccination research in other resource-constrained regions like Tanzania [43] and Senegal [44].

Thus, after piloting the study questionnaire, we changed the data collection platform from google forms to ODK, allowing participant data to be collected offline and uploaded to the database whenever internet connectivity was available. We efficiently used multiple languages based on participants’ preferences.

A debriefing meeting about the study was held with the health facility staff, teachers, and community healthcare workers to disseminate information regarding this research. Study participants were sampled from community settings with the help of community healthcare workers. Interviews were conducted in a quiet and private place convenient for the participant, such as community halls. After the interviews, participants were given educational materials on HPV vaccines obtained from the Ministry of Health to bridge the knowledge gap. In addition, participants were allowed to give feedback about the interviews at the end of each session.

### 2.4. Data Management and Analysis

We used password-locked Android-based mobile tablets to collect and submit data to an online Ona server. Data collection was managed using ODK Aggregate, an intermediate platform for the server and later downloaded into Excel. Data collection was monitored daily by the principal investigator, checked for consistency, cleaned, transferred, and analysed in STATA version 17/BE (Stata Corp., College Station, TX, USA). All analyses accounted for the clustering of respondents within the zones using robust estimation of standard errors (which was achieved using the Stata commands to analyse survey data). In addition, the analysis accounted for the stratification by sub-districts within Lusaka province.

The item scores were summed to create a score for six HBM constructs scales and one knowledge scale. The means and standard deviations or median and interquartile ranges were used as appropriate to summarise continuous variables (age, the six HBM construct scores and knowledge score) and report whether the respondent consented to vaccinate their daughter.

Simple logistic regression models were fitted with robust estimation of standard errors with “consent to vaccinate daughter” as the response variable and one of the predictor variables at a time, to assess for any association between the predictor variable and consent to vaccinate daughter. Afterwards, a multivariable logistic regression model was fitted with six HBM construct scores (priori), and other variables found significant at a 20% level from the univariable logistic regression model. Variables were dropped from the multivariable model sequentially until only important variables remained. Finally, interactions between six HBM construct scores and modifying variables that remained in the final model were considered individually. The Hosmer Lemeshow test was done to assess the goodness of the model fit. The odds ratios are presented with 95% confidence intervals.

Using principal component analysis, we used household assets and other related characteristic variables to calculate the wealth index as a proxy for socio economic status [26]. The wealth index was categorised into five social economic quintiles (poorest, poorer, middle, richer, richest) [28] and further dichotomised (poorest, poorer, middle = poor, richer, richest = rich) for analysis owing to the small sample size.

Further, generalised structural equation modelling was conducted to understand the interrelationships between variables and assess mechanisms of association [45]. Direct, indirect, and total effects were studied to understand the mechanism through which knowledge affected parental consent to vaccinate daughters, part of which could occur through the HBM constructs. The effect of knowledge (exposure variables) on parental consent to vaccinate daughters (outcome variable) while controlling for HBM constructs was the direct effect, while the indirect effect occurred because knowledge affects the HBM constructs, which in turn affect parental consent to vaccinate daughters. Direct and indirect effects together formed the total effects on the outcome. All models independently adjusted for other co-variates, including age, sex, socio-economic status, marital status, and education levels. Odds ratios were used to estimate measures of effect. Bootstraps (52) replications were used to compute standard errors for the effects estimates [45].

The writing of this manuscript was guided by the STROBE statement checklist [46].

## 3. Results

We enrolled 400 participants; 215 (53.8%) reported consenting to the HPV vaccine for their daughters, and all reported that their daughters received it. The demographic characteristics and total knowledge scores are summarised in Table 1.

Health Belief Model constructs scores according to parental consent to vaccinate their daughter.

**Figure 2:**
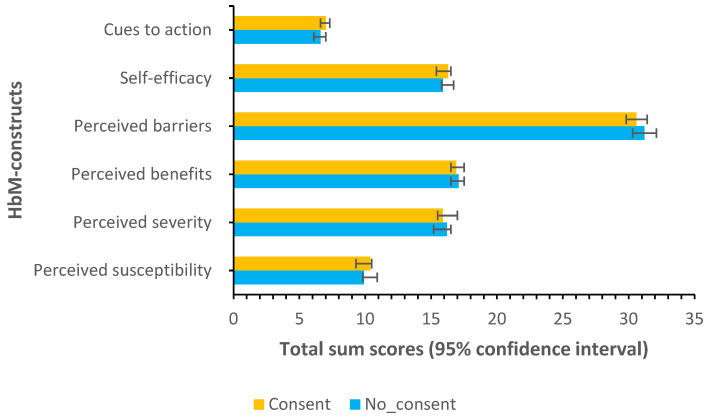
HBM constructs scores according to consent to vaccinate daughter: error bars are 95% confidence intervals.

Figure 2 shows that consenting and non-consenting participants had similar mean scores for all HBM constructs.

### 3.1. HPV and HIV-Related Characteristics

A higher proportion of female participants who consented to vaccinate their daughter had screened for cervical cancer compared to non-consenting parents. Participants who knew their HIV status as positive, knew someone with cervical or HPV-related cancers or someone who has had genital warts were more likely to consent to daughters’ vaccination Table 2.

### 3.2. Correlates of Parental Consent to Vaccinate Daughter

In the univariable logistic regression model, we found that wealth index, knowledge score, knowing someone with cervical cancer or other HPV-related cancers, knowing someone with genital warts and living with HIV were associated with providing consent to vaccinate the daughter. When adjusting for variables that were significant at a 20% level in the univariable model, the multivariable logistic regression showed that HPV/HPV vaccine knowledge, ever knowing someone with HPV-related cancer and none of the HBM construct scores were independently associated with consent to vaccinate the daughter (Table 3).

### 3.3. Mediation Effect of Knowledge on Parental Consent for HPV Vaccination

Mediation analysis was performed to explore and assess if HBM constructs are a mechanism through which knowledge levels affect parental consent choices. Knowledge levels were significantly associated with parental consent as a direct effect; however, the indirect effects of knowledge (mediated through HBM constructs) were not significant (Table 4). All other covariates: socio-economic status, marital status, and education level, were not significantly associated with parental consent to vaccinate their daughters or any of the HBM constructs.

## 4. Discussion

This study found that over half of our sample participants provided parental consent for the daughters’ HPV vaccination. We framed our study within the HBM based on its usefulness in exploring vaccination behaviour. However, we did not find any significant associations between HBM constructs and parental consent for the daughter’s HPV vaccine uptake, associated factors included; having screened for cervical cancer, knowing someone with HPV-related conditions, socio-economic status and marginally for those living with HIV.

Parental consent to daughters’ HPV vaccination was 53.8%, and their daughters vaccinated, giving an uptake of 53.8% amongst our sample. This uptake is higher than the 39% [19] coverage reported during the same period at the national level. This could be because our data is regional and may not be generalized countrywide. However, 53.8% HPV vaccine uptake is still lower than the recommended 70% for population herd immunity to be achieved [6]. Additionally, the girls’ attitudes may affect HPV vaccine uptake, which could be influenced by myths, misinformation, and parental refusal [18].

Parental consent plays a crucial role in HPV vaccination as daughters believe and act upon what their parents tell them. An earlier hypothetical study done in Lusaka, Zambia, before the HPV vaccination demonstration project (2013–2017) and national rollout of the HPV vaccination (2019) showed that 100% of the parents intended to vaccinate their daughters or themselves [23]. On the contrary, another study in Lusaka, Zambia, after the HPV demonstration project, reported that only 6.5% of parents had vaccinated their daughters [35]. These studies show an extremely stark contrast to our finding, which could be because we reported parental consent and actual HPV vaccine uptake other than intent in a broader national HPV vaccination program. Yu et al. [47] reported similar findings among African American parents with a similar sample size. Our results on parental consent are below the expected average, this could be explained by limited knowledge of HPV and HPV vaccine among parents and the volumes of circulating myths and misinformation about vaccines in general and the HPV vaccine, accentuated by the COVID-19 pandemic [19]. Information about the current national HPV vaccine coverage shows a drop from 75% in 2021 to 39% in 2021 [19].

We did not find any differences in HBM construct scores between consenting and non-consenting parents, as earlier hypothesized. Similarly, Vermandere and others found no differences regardless of the daughter’s vaccination in a selected cohort of parents in Kenya [28]. However, there are other studies which have contrasting results in different regions. For example, Krawcyzk et al. [29], in a study done in Canada, found that perceived severity, perceived susceptibility, cues to action and low perceived barriers were associated with parental vaccination of adolescent children. Another study in Nigeria reported that cues to action, such as recommendations by healthcare providers and friends/relatives, predicted HPV vaccination [48].

The plausible explanation could be that we had low awareness of HPV and HPV vaccine in our cohort, and people are unlikely to commit to or have firm perceptions/beliefs about what they do not know. Therefore, people empowered with knowledge may be able to give a well-informed position. Further, inherent within the HBM framework and previous literature, the constructs’ relationship was assumed linear without variable ordering, which is considered a more complex approach [49].

Combined high knowledge of HPV and HPV vaccine was associated with vaccination in univariate analysis. However, this was not significant after adjusting for possible confounders. Similarly, in mediation analysis, knowledge was a statistically significant direct factor associated with parental consent for daughters, but not indirectly through the HBM constructs. Knowledge plays a key role, as low levels may predispose one to hold fast to myths and misinformation. In other studies, parental knowledge is highly associated with the willingness to vaccinate daughters [34,35,50]. Like our findings, a study in Sweden did not find any differences in knowledge between consenting and non-consenting parents [51].

The possible explanation is that the HPV vaccine is relatively new in Zambia, despite an earlier demonstration project; hence some parents may not be knowledgeable about it. Further, the HPV vaccination program is campaign-based, and mass media messages and other activities to raise awareness only occur briefly before and during the activity. Therefore, it is important to consider ongoing messaging that can be reinforced during vaccination [52].

This study revealed that the rich were more likely to consent to the HPV vaccine than the poor. Similarly, a study in Uganda based on demographic health data found that those in the middle wealth quintile were more likely to vaccinate their daughters [53]. Another study showed that people with a low SES are less likely to initiate and complete vaccination due to a delay in receiving and comprehending health messages [54]. Notwithstanding, people with low SES yet are most affected but continue with low uptake of health interventions such as the HPV vaccine.

We found that HIV-positive parents are more likely to vaccinate their daughters in univariable and marginally in multivariable analyses. There has been an emphasis on cervical screening WLHIV, especially in high-burdened regions like Zambia, to reduce the burden of cervical cancer [55]. As such, cervical cancer screening and education are intensified to increase WLHIV’s uptake of screening services [56]; hence more are likely to know about other preventative measures like HPV vaccination.

Therefore, it is not surprising that WLHIV and those who have screened for cervical cancer are more likely to consent to their daughter’s vaccination based on their exposure to information on HPV and the HPV vaccine’s benefits. Wigfall et al. [57] reported that among WLHIV, those who were aware that HPV caused cervical cancer were more likely to be aware of the HPV vaccine. Further, women screened for cervical cancer were more likely to consent to their daughter’s HPV vaccination. An earlier study in Zambia reported similar findings that cervical cancer screening was significantly associated with daughters’ HPV vaccination [35].

## 5. Strengths and Limitations

This is one of the first studies in Zambia regarding parental consent for the HPV vaccine since the national rollout, framed in a well-recognised model. Even though the data is regional and collected from the capital city, the study can serve as a pilot, which can be scaled up to get a nationally representative sample. Using the health belief model is important as understanding results framed in theories is easier.

The HBM has been widely used as a cognitive theory; hence this study adds evidence to the existing literature on circumstances where the behaviour of interest, as in this case, may not be directly influenced by the HBM constructs [49]. Further, our use of the generalized structural equation model overcomes the limitations of the standard structural equation model, which assumes a normal distribution of variables [45]. Additionally, we could simultaneously compare the direct and indirect effects of multiple interacting factors.

This study is without limitations; firstly, it was done in the Lusaka district hence the results may not represent the rest of the country, affecting the external validity. Secondly, recall bias could exist as some adolescents’ vaccination period was as far back as two years. However, parents usually have a good memory for a child’s health-related issues. Thirdly, girls could have received the vaccine without parents knowing since the current approach is opt-out, and the girl’s desire is considered primarily. Therefore, these girls may have been reported as not-vaccinated by parents when the converse was true. Fourth, non-consenting parents may have reported consenting for their daughters due to social desirability since the questionnaire was interviewer-administered.

Fifth, this data was collected during the COVID-19 pandemic when there was a lot of mistrust around the COVID-19 vaccine, which could have significantly heightened the barriers in both groups during our data collection. Finally, the data was collected when the behaviour of interest, HPV vaccination, had already occurred. Therefore, the hypothesis can only be adequately tested where beliefs are known to have existed before the behaviour they are to determine; otherwise, individuals’ perceptions could be modified in areas relevant to HPV vaccination. There is the potential that vaccination status may have led to selective exposure or recall bias, thus suggesting reverse causality.

## 6. Conclusions

This study has highlighted modifiable correlates of parental consent for adolescent HPV vaccination, which include socio-economic status, cervical cancer screening uptake and knowledge. Equity in vaccination programs should be prioritised as the rich are more likely to consent and get the vaccine and more likely to have screened for cervical cancer.

Focus on raising community awareness on the role of HPV vaccination in preventing cervical cancer, especially among those from low socio-economic status, should be a priority to improve future programming and uptake. Equitable access to the provision of the HPV vaccine is essential to reduce the gap between the rich and the poor. Further, education on the relationship between HPV and cervical cancer and the preventive role of HPV vaccination should be integrated into the screening programs. This approach may improve knowledge levels among parents. Additionally, at the policy level, the feasibility of having ongoing HPV vaccination should be explored as it may improve access; Zambia can take a leaf from countries like Senegal [44].

## Figures and Tables

**Table 1:** Socio-demographic characteristics and knowledge scores of participants ^a^.

Variable	Total Sample N = 400 (%)	Parental Consent to Vaccinate a Daughter	
		No, *n* = 185	Yes, *n* = 215
Age years mean [95% CI]	45.7 [44.3–47.1]	46.3 [44.1–48.6]	45.3 [43.6–47.1]
Sex			
Male	50 (12.5)	24 (13.1)	26 (12.0)
Female	350 (87.5)	161 (87.0)	189 (87.9)
Marital status			
Married	264 (66.0)	121 (65.6)	143 (66.5)
Single	136 (34.0)	64 (34.5)	72 (33.5)
Education level			
None/primary	181 (45.3)	89 (48.1)	92 (42.8)
Secondary/Tertiary	219 (54.8)	96 (51.9)	123 (57.2)
Employment			
Unemployed	330 (82.8)	160 (86.5)	170 (79.1)
Employed	70 (17.3)	25 (13.5)	45 (20.9)
Daughter goes to school			
Yes	352 (88.0)	157 (84.8)	195 (90.7)
No	48 (12.0)	28 (15.1)	20 (9.3)
Wealth index			
Poorest/poorer/middle	245 (61.3)	124 (67.0)	121 (56.3)
Richer/richest	155 (38.8)	61 (33.0)	94 (43.7)
Total HPV/HPV vaccine knowledge mean score	11.1 [10.0–12.2]	9.0 [7.4–10.7]	12.7 [11.1–14.3]

Key: 95% CI-95% confidence interval, wealth index α = 0.74, Kaiser-Meyer-Olkin = 0.81.

**Table 2:** HPV and HIV-related characteristics of participants ^a^.

Characteristic	Total Population N = 400 (%)	Parental Consent to Vaccinate a Daughter		*p*-Value
		No, *n* = 185 (%)	Yes, *n* = 215 (%)	
Ever been screened for cervical cancer? *n* = 350				0.001
Yes	247 (70.6)	99 (61.5)	148 (78.3)	
No	103 (29.4)	62 (38.5)	41 (21.7)	
Know someone with cervical cancer?				0.370
Yes	167 (41.8)	72 (39.3)	95 (43.8)	
No	233 (58.3)	111 (60.7)	122 (56.2)	
Know someone with other HPV-related cancer, e.g., vulva, penis, oral				0.011
Yes	43 (10.8)	12 (6.6)	31 (14.3)	
No	357 (89.3)	173 (93.5)	184 (85.6)	
Know someone with genital warts?				<0.001
Yes	85 (21.3)	23 (12.4)	62 (28.8)	
No	315 (78.8)	162 (87.6)	153 (71.2)	
Intent to vaccinate other daughters				0.843
No	31 (7.8)	15 (8.1)	16 (7.4)	
Yes	291 (72.8)	132 (71.4)	159 (74.0)	
Not sure	78 (19.5)	38 (20.5)	40 (18.6)	
Daughter’s HIV status? *n* = 319				0.193
Negative	311 (97.5)	127 (96.2)	184 (98.4)	
Positive	8 (2.5)	5 (3.8)	3 (1.6)	
HIV status *n* = 320				0.036
Negative	200 (62.5)	94 (69.1)	106 (7.6)	
Positive	120 (37.5)	42 (30.9)	78 (42.4)	

^a^ Values are given as a number (percentage) unless indicated otherwise, N = 400 unless otherwise stated. Wald test was used to calculate *p*-values.

**Table 3:** Adjusted associations between predictors and parental consent to vaccinate daughter.

Variable	COR [95% CI] ^a^	*p*-Value	AOR [95% CI]	*p*-Value
Socio-demographics				
Age (years)	0.99 [0.97–1.01]	0.413		
Sex				
Male	Ref			
Female	0.54 [0.28–1.03]	0.063		
Marital status				
Married	Ref			
Single	1.04 [0.62, 1.72]	0.890		
Education				
None/Primary	Ref			
Secondary/Tertiary	1.07 [0.64–1.73]	0.789		
Employment				
Employed	Ref			
Unemployed	1.49 [0.76–2.91]	0.245		
HPV/HPV vaccine knowledge mean score	1.04 [1.01–1.06]	0.004		
Wealth index				0.005
Poorest/poorer/middle	Ref		Ref	
Richer/richest	1.93 [1.16–3.21]	0.011	2.32 [1.29–4.16]	
Health Belief Model constructs				
Perceived susceptibility score	1.04 [0.97, 1.12]	0.241	1.05 [0.96–1.14]	0.262
Perceived severity score	0.97 [0.91, 1.04]	0.486	0.98 [0.91–1.06]	0.590
Perceived benefits score	0.99 [0.90–1.09]	0.835	1.06 [0.94–1.19]	0.321
Perceived barriers score	0.98 [0.94–1.02]	0.313	0.96 [0.90–1.01]	0.108
Self-efficacy	1.04 [0.96–1.12]	0.356	0.98 [0.88–1.09]	0.726
Cues to action	1.08 [0.98–1.20]	0.128	1.05 [0.92–1.19]	0.492
HPV and HIV-related characteristics				
Ever known anyone with cervical cancer	1.14 [0.70, 1.86]	0.588		
Ever known anyone with HPV-related cancers	2.60 [1.22, 5.55]	0.013		
Ever screened for cervical cancer	2.32 [1.43, 3.80]	0.001	1.93 [1.03–3.62]	0.041
Ever known anyone with genital warts	2.54 [1.32–4.87]	0.005	2.23 [1.04–4.76]	0.039
Daughters’ HIV status				
Negative	Ref			
Positive	0.64 [0.13, 3.01]	0.567		
Parental HIV status				0.078
Negative	Ref		Ref	
Positive	1.93 [1.09, 3.42]	0.024	1.07 [0.01–1.36]	

Note: COR-crude odds ratios, AOR-adjusted odds ratios. ^a^ Values are given as a number (percentage) unless indicated otherwise

**Table 4:** Mediation effect of knowledge on parental consent for HPV vaccination.

	Mediators							
	Perceived Barriers		Perceived Susceptibility		Perceived Severity		Perceived Benefits	
Effects	OR [95% CI]	*p*-Value	OR [95% CI]	*p*-Value	OR [95% CI]	*p*-Value	OR [95% CI]	*p*-Value
Total	2.37 [1.62–3.47]	<0.001	2.39 [1.58–3.61]	<0.001	2.39 [1.45–3.93]	0.001	2.38 [1.53–3.71]	<0.001
Indirect	0.98 [0.95–1.01]	0.297	1.01 [0.97–1.04]	0.777	1.0 [0.97–1.03]	0.901	1.0 [0.97–1.03]	0.993
Direct	2.42 [1.66–3.52]	<0.001	2.37 [1.57–3.59]	<0.001	2.38 [1.45–3.90]	0.001	2.39 [1.52–3.74]	<0.001

All models independently adjusted age, sex, SES, marital status, and education levels. Bootstraps (50) replications were used to compute standard errors for effects estimates.

## Data Availability

The data presented in this study are available on request from the corresponding author. The data are not publicly available due to ethical issues as it is part of a PhD in progress.
